# Cancer stem cell property and gene signature in bone-metastatic Breast Cancer cells

**DOI:** 10.7150/ijbs.45693

**Published:** 2020-07-19

**Authors:** An Luo, Yue Xu, Shujun Li, Jinxia Bao, Jinhui Lü, Nan Ding, Qian Zhao, Yuting Fu, Fei Liu, William C. Cho, Xunbin Wei, Haiyun Wang, Zuoren Yu

**Affiliations:** 1 Research Center for Translational Medicine, Shanghai East Hospital, School of Life Sciences and Technology, Tongji University, Shanghai 200120, China.; 2 Department of Bioinformatics, School of Life Sciences and Technology, Tongji University, Shanghai, China.; 3 Department of Gastroenterology, Shanghai East Hospital, Tongji University, Shanghai 200120, China.; 4 State Key Laboratory of Oncogenes and Related Genes, Shanghai Cancer Institute, Med-X Research Institute and School of Biomedical Engineering, Shanghai Jiao Tong University, Shanghai, China.; 5 Biomedical Engineering Department, Peking University, Beijing, China.; 6 The Third Hospital of BaoGang Group, Baotou, China.; 7 Department of Clinical Oncology, Queen Elizabeth Hospital, Kowloon, Hong Kong, China.

**Keywords:** bone metastasis, triple-negative breast cancer, cancer stem cell, malignancy

## Abstract

The majority of the deaths from breast cancer is due to metastasis. Bone is the most common organ to which breast cancer cells metastasize. The mechanism regulating the bone-metastatic preference remains unclear; there is a lack of a gene signature to distinguish bone-metastatic breast cancer cells. Herein, florescence-labeled MDA-MB-231 cells were transplanted into the fat pads of of the mammary gland in nude mice to generate breast tumors. Tumor cells invaded into the circulation were tracked by *in vivo* flow cytometry system. Metastatic tumor cells in the bone were isolated using fluorescent-activated cell sorting technique, followed by assays of cell colony formation, migration and invasion, mammosphere formation *in vitro*, mammary gland tumorigenesis *in vivo*, and Next-Generation Sequencing analysis as well. Through tumor regeneration and cell sorting, two bone-metastatic cell sublines were derived from MDA-MB-231 cells; which showed higher abilities to proliferate, migrate, invade and epithelial-to-mesenchymal transit *in vitro*, and stronger ability to regenerate tumors and metastasize to the bone *in vivo*. Both cell sublines exhibited cancer stem cell-like characteristics including higher expression levels of stem cell markers and stronger ability for mommaspheres formation. Furthermore, a Normal Distribution-like pattern of the bone-metastatic cells invading into circulation was firstly identified. Deep-sequencing analysis indicated upregulation of multiple signaling pathways in regulating EMT, cell membrane budding and morphologic change, lipid metabolism, and protein translation, which are required to provide adequate metabolic enzymes, structural proteins, and energy for the cells undergoing metastasis. In conclusion, we established two bone-metastatic breast cancer cell sublines, carrying higher degree of stemness and malignancy. The gene signature distinguishing the bone-metastatic breast cancer cells holds therapeutic potentials in prevention of breast cancer metastasis to the bone.

## Introduction

Breast cancer is the most common cause of cancer death among women around the world 1. Although having traditional therapies including radiotherapy, chemotherapy and hormone therapy, breast cancer metastasis still remains incurable due to limited understanding of the molecular mechanisms governing cancer metastasis and relapse. As a result, the 5-year survival rate of patients is only approximately 27% for metastatic breast cancer 2.

Breast cancer is classified to luminal, basal-like, ERBB2, and normal breast-like subtypes, in which basal-like tumors show the highest degree of malignancy, the poorest outcomes and the lowest rate of 5-year survival. Around 80% of basal-like breast cancers are characterized as triple-negative breast cancer (TNBC) because of lacking the expression of estrogens receptor (ER), progesterone receptor (PR) and human epidermal growth factor receptor 2 (HER2), representing ~10-20% of all breast cancer cases 3. Since these receptors often served as targets for hormone therapy, TNBC patients do not respond to the typical treatment. The limited effects of combination therapies with chemotherapeutic drugs make TNBC aggressive, invasive, metastatic and incurable.

Metastasis of cancer is a multi-step process including cancer cell invasion from tumors at primary site, intravasation through lymph or blood vessels, survival in the circulation, extravasation into a distant site, and colonization 4,5. The most common organ breast cancer metastasize to is the bone, followed by the lung, liver and brain 6. About 70% of patients with advanced breast cancer have metastasis to the bone 7, which is associated with poor prognosis and reduced life expectancy. The patients with bone metastasis suffer severe pain in the late-stages mainly caused by mechanical pressure from the metastatic tumor cell population, and inflammatory cytokines released by the tumor cells and the surrounding bone pores 8. In addition, restrained mobility, hypercalcemia, fracture, spinal cord compression, and/or decreased bone marrow regeneration ability caused by bone metastasis seriously affect the quality of life in patients with advanced breast cancer.

It is believed that cellular heterogeneity within a tumor endows a small population of tumor cells with augmented metastatic abilities. In most cases, metastasis from the primary site to a distant secondary site is selective upon tumor type and/or host organ microenvironment 9. The subset of metastatic cell population in the primary tumors may had already contained a gene signature that is predictive of distant metastasis and poor survival 10. So the microenvironment in the bone must provide the most suitable “soil” for the growth of breast tumor cell “seed” which may hold certain properties, such as stronger attachment to the bone than to other sites, and/or better resistance to immune system in the bone. Furthermore, the enriched blood vessels in the bone 11 and the overexpression of bone metastasis genes including interleukin-11 and CTGF 10 attract breast cancer cells to home and thrive at the bone.

Although thousands of publications have reported factors involving in regulation of cancer metastasis, how metastatic tumor cells select host organs remains an open question. It is still largely unknown and is a subject of debate for the mechanisms determining the metastatic cell fate. Drawing inspiration from literature that transplantation of metastatic tumor cells to mice can lead to enrichment and augment in metastasis 10, 12, 13, we established a bone-metastatic triple-negative breast cancer cell subline by transplanting a widely-used cell line, MDA-MB-231, to the fad pat of female nude mice to grow tumors *in vivo*. The bone metastatic cell sublines exhibited higher rate of proliferation, migration, invasion and bone metastasis. In addition, the cell sublines expressed higher levels of stem cell markers including BMI-1, Nanog and OCT4, and formed mammospheres with increased number and size, compared to the parental MDA-MB-231 cells. Deep sequencing analysis indicated upregulation of genes involved in the metastasis-related signaling pathways, such as EMT, cell morphologic change, and protein translation and secretion. In particular, upregulation of lipid metabolism genes was found in the bone-metastatic cells, indicating the energy source drives breast cancer cell metastasis to the bone. In conclusion, the cell sublines we established not only provide useful cell models for determining the mechanism regulating bone metastasis of breast cancer cells, but also shed light on strategy development in treatment of bone metastatic breast cancer.

## Materials and Methods

### Animals

Animal studies were approved by the Institutional Animal Care and Use Committee of the Tongji University School of Medicine. 6-week-old female nude mice were purchased from the Silaike Animal Company (Shanghai, China).

### Cell lines and cell culture

Human breast cancer cell line MDA-MB-231 and package cell line HEK293T were purchased from ATCC (Manassas, VA, US) and maintained in our laboratory. Cells were cultured in DMEM medium containing penicillin and streptomycin (100 mg/L) and 10% fetal bovine serum (FBS) at 37 °C in a humidified environment with 5% CO2. The medium was refreshed every 2 days. Cells were performed for functional assays at 70-80% confluent.

### Generation of RFP-labeled MDA-MB-231 cell line

The TurboRFP-carrying lentiviral vector pLemiR (Open biosystems) and packaging plasmids psPAX2 (http://www.addgene.org/12260/) and pMD2.G (http://www.addgene.org/12259/) were co-transfected into HEK293T cells. In 48 hours, the lentiviruses in supernatant were collected to infect MDA-MB-231 cells using Polybrene (final concentration 8 μg/ml). After puromycin selection for 2 weeks, MDA-MB-231 cells stably expressing RFP florescence were generated. RFP measurement was applied to determine the transfection and infection efficiency.

### Preparation of breast tumor burden mice

5x10^5^-1x10^6^ RFP-MDA-MB-231 cells were mixed with matrigel and injected into the fat pad of the fourth mammary gland of nude mouse. From day 10 on, the volume of tumors was measured every other day until sacrifice when the diameter of tumor reached ~10 mm.

### Isolation of bone metastatic MDA-MB-231 cells

Immediately after sacrifice of breast tumor burden mouse, femora and shinbone from hind legs were collected and washed with 1×PBS. Opening the bones using a scissor at the position of joints, and flushing the bone marrow out using a syringe to cell culturing medium. After rinse and wash with fresh medium, the cells in bone marrow were cultured for 1-2 weeks. The RFP-positive cells were examined with a fluorescence microscope (Leica, Germany), sorted using flow cytometry (BD Biosciences, USA), and cultured for proliferation.

### Establishment of bone-metastatic cell sublines

The RFP-MDA-MB-231 cells isolated from the bone of breast tumor burden mouse were purified and amplified *in vitro*, followed by transplantation into the fat pad of the fourth mammary gland of nude mouse to regenerate breast tumors, and re-isolate RFP-positive cells from the bone marrow. After three rounds of this way for breast tumor generation and bone metastasis, the bone-metastatic cell sublines BM1 and BM2 were established from different individual mouse, respectively.

### siRNA transfection

siRNAs to Foxg1, Trem1, Slpi and negative control (NC) were purchased from Guangzhou RiboBio Co., Ltd (Guangzhou, China). The HiPerFect transfection reagent from Qiagen (Venlo, The Netherland) was used for RNA oligos cell transfection according to the manufacturer's instructions.

### Quantitative real-time PCR analysis

Total RNA was extracted with Trizol reagent (Invitrogen, US) from cells. Regular approach and random primer were used for reverse transcription. The SYBR Green Master Mix (Applied Biosystem, Thermo Fisher Scientific) and 7900 HT Sequence Detection System (Applied Biosystem, Thermo Fisher Scientific) were used for real-time PCR assays. β-actin and GAPDH were used for normalization.

### Cell proliferation assays

Cell Counting Kit-8 (CCK-8) assays were applied for determining cell proliferation. Briefly, 1×10^4^ cells/well were seeded into 96-well plate. DMEM medium was set as blank control. After culturing for 0, 24 and 48 hours as indicated, each well was added with 10 μl CCK-8 solution, and then cultured for 3 hours at cell-culturing condition followed by measurement of OD value at 450 nm wavelength.

### Western blot

Cell lysates were measured for protein concentration using BCA kit (Beyotime Biotechnology, Shanghai.). 50 μg of lysates was separated by 10% SDS/PAGE. The proteins were transferred to nitrocellulose membrane. After being blocked in 5% milk (w/v) at room temperature for 1 hour, the membranes were incubated at 4 °C overnight with primary antibodies (1:1,000). Following 1×PBST washing, the membranes were incubated with secondary antibodies (1:3,000) at room temperature for 1 hour followed by ECL staining. The following antibodies were used: anti-Snail1 (sc-271977, Santa Cruz Biotechnology), anti-Slug (sc-166902, Santa Cruz Biotechnology), anti-ZEB1 (sc-515797, Santa Cruz Biotechnology ), anti-ZEB2 (sc-271984, Santa Cruz Biotechnology), anti-Twist (sc-81417, Santa Cruz Biotechnology), anti-DKK1 (sc-374574, Santa Cruz Biotechnology), anti-BMI-1 (sc-390443, Santa Cruz Biotechnology), anti-Oct4 (2750S, Cell Signaling Technology), anti-Nanog (4903S, Cell Signaling Technology), anti-Vimentin (sc-32322, Santa Cruz Biotechnology), anti-GAPDH (5174, Cell Signaling Technology), and anti-β-actin (sc-47778, Santa Cruz Biotechnology). HRP-conjugated anti-rabbit IgG (7074S, Cell Signaling Technology) and HRP- conjugated anti-mouse IgG (7076S, Cell Signaling Technology) were used as secondary antibodies.

### Colony formation assay

Cells were plated in a 12-well plate with 500 cells per well, and cultured under regular condition for two weeks. The colonies were fixed with 4% paraformaldehyde, and stained with 0.5% crystal violet solution for 30 min. The colonies with diameter over 40 μm were counted under microscope for quantitative analysis.

### Wound healing assay

Cells were plated in 6-well culture plates to achieve over 90% confluence. A vertical wound per well was created using a 10 µL pipette tip. The cells were cultured in DMEM medium containing 0.1% FBS. Images were captured at the indicated time to assess the wound closure rate.

### Cell invasion assay

Transwell chambers with 8 μm pores (Corning, USA) were pre-coated with ECM Gel (E1270, Sigma-Aldrich, USA), and placed in a 24-well plate containing DMEM medium supplemented with 10% FBS and 50 μg/mL fibronectin. 2×10^4^ cells were seeded in the chamber with serum-free medium, followed by 6-hour's incubation at 37 °C and 5% CO2. Cells adherent to the upper surface of the chambers were removed using a cotton applicator. Chambers containing invaded cells were stained with 0.4% violet crystal acetate overnight. The stained cells were photographed for quantitative analysis.

### Mammosphere formation assay

Cells were plated at a density of 2,000 cells/well in 12-well ultra-low adherent cell culture plate (Corning, USA) and grown in DMEM/F12 containing 1x B27 supplement (Invitrogen), 20 ng/mL human epidermal growth factor (EGF; Sigma) and 20 ng/ml of human basic fibroblast growth factor (bFGF; R&D Systems) for 7-14 days without disturbing the plate. After culturing, the mammospheres with diameter greater than 40 µm were counted under a microscope for quantitative analysis.

### *In vivo* flow cytometry (IVFC) analysis

IVFC was applied for the real-time detection of BM1 and BM2 cells in circulation. Briefly, tumor-burden mice were anesthetized and placed on the flow cytometry platform. The major arteries of the mouse ear were visualized under illumination with a 535±15 nm light emitting diode (LED) using a charge-coupled device (CCD) camera. An artery with a diameter of 50 µm was chosen for data acquisition. The 561-nm laser was modulated into a slit-shaped beam using a cylindrical lens for the laser excitation. This laser slit was positioned across the selected artery. The length and width of the laser slit at the focal plane were approximately 72 µm and 5 µm, respectively. The RFP signaling in cells would be excited when the cells passed through the laser slit. The emitted fluorescence was collected by a photomultiplier tube (PMT) and digitized with a data acquisition card at a sampling frequency of 5 kHz. The detection was performed at week 2, week 3 and week 4 after cell transplantation. Each mouse was detected for continuous 30 min each time.

### *In vivo* imaging system

BM1 and BM2 cells were transduced with a lentiviral vector expressing firefly luciferase to establish stable cell lines, followed by a tail vein injection with 1×10^6^ cells in 100 μl of PBS to each nude mouse. The substrate luciferin was applied through intraperitoneal injection at a dose of 150 mg/kg body weight around 5 minutes before measurement. Images were collected for 120 seconds using the *In vivo* imaging system (NightOWL LB 983, Berthold, Germony). Florescence intensities in the bone and lung regions were quantified using IndiGO™ software.

### RNA deep-sequencing

Total RNAs from BM1, BM2 and control cells were applied for whole transcriptome sequencing in triplicates (BGI Genomics, China). Briefly, cDNA library was prepared using N6 random primer and PCR amplification. Reads were cleaned using SOAPnuke software (BGI Genomics, China) by removing those reads with low quality tags, contamination formed by adaptor-adaptor ligation and high rate of N nucleotides. The quality of the clean reads was evaluated with FastQC. The paired-end reads were aligned to the human reference Ensembl Version GRCh38.91 using the splice-aware aligner STAR (v2.4.0j). The abundance of each gene was quantified as TPM (Transcripts per million) value, which was evaluated by a statistical method RSEM (RNA-Seq by Expectation Maximization). To obtain correct statistical inference, batch effects were removed by svaseq (Leek, 2014). Afterwards, the differentially expressed genes (DEGs), defined by fold change (FC) ≥ 2 and a false discovery rate (FDR) < 0.05, were called using the DESeq2. A scatterplot of the DEGs were drawn via the “ggplot2” package in the R platform. The number of reads for each sample was shown in Supplemental Table S1.

### Hierarchical clustering and principal component analysis

We calculated the standard deviation (SD) of each gene across samples and selected those with SD ≥ 1 to generate a hierarchical clustering with the “pheatmap” package in R. Principal Component Analysis (PCA), an unsupervised learning technique, was used to generate 1^st^, 2^nd^, and 3^rd^ principal component. The samples were clustered based on three principal components and distributed in three-dimensional (3D) space by the “Scatterplot3d” package in the R platform.

### Epithelial Mesenchymal Transition (EMT) score

EMT score was designed to evaluate the occurrence of EMT process by using EMT signature genes 14. Briefly, it was calculated as the mean expression of epithelial markers subtracted from the mean expression of mesenchymal markers. EMT signature encompasses a set of core EMT genes that have molecular alterations at the protein level, in particular, the epithelial markers include Collagen IV alpha 1 (COL4A1), Basal Cytokeratins (KRT5 and KRT14), Luminal Cytokeratins (KRT8 and KRT18), Desmoglein-3 (DSG3), E-Cadherin (CDH1), Laminin (LAMA1, LAMA2, LAMA3, LAMB1, LAMB3 and LAMC1), MUC-1 (MUC1) as well as Syndecan-1 (SDC1), whereas the mesenchymal markers include Alpha-SMA (ACTA2), Fibronectin (FN1), N-cadherin (CDH2), S100A4 (S100A4), Slug (SNAI2), Snail (SNAI1 and SNAI3) as well as Vimentin (VIM). Higher EMT score correlates with a mesenchymal expression pattern.

### Gene Set Enrichment Analysis (GSEA)

GSEA was employed to determine the gene sets, including from KEGG, Gene Ontology (GO), Cancer Hallmarks, and Reactome databases, enriched by a pre-ranked list of all genes, which were sorted by the statistical significance of differential expression defined by DESeq2 analysis. Among 5,160 gene sets in this analysis, 50 were cancer hallmark gene sets, which summarize and represent specific well-defined biological states or processes and display coherent expression 15, 4,436 were from Biological Process of Gene Ontology (GO(BP)) terms (http://geneontology.org/), and 674 were from Reactome (http://www.reactome.org/). Gene sets with FDR < 0.05 were statistically significant.

### Statistical analysis

Data are presented as mean ± SEM unless stated otherwise. The standard two-tailed student's *t*-test was used for statistical analysis, in which p<0.05 was considered significant.

## Results

### Establishment of bone-metastatic cell sublines BM1 and BM2 from triple negative breast cancer cell MDA-MB-231

**Figure 1:**
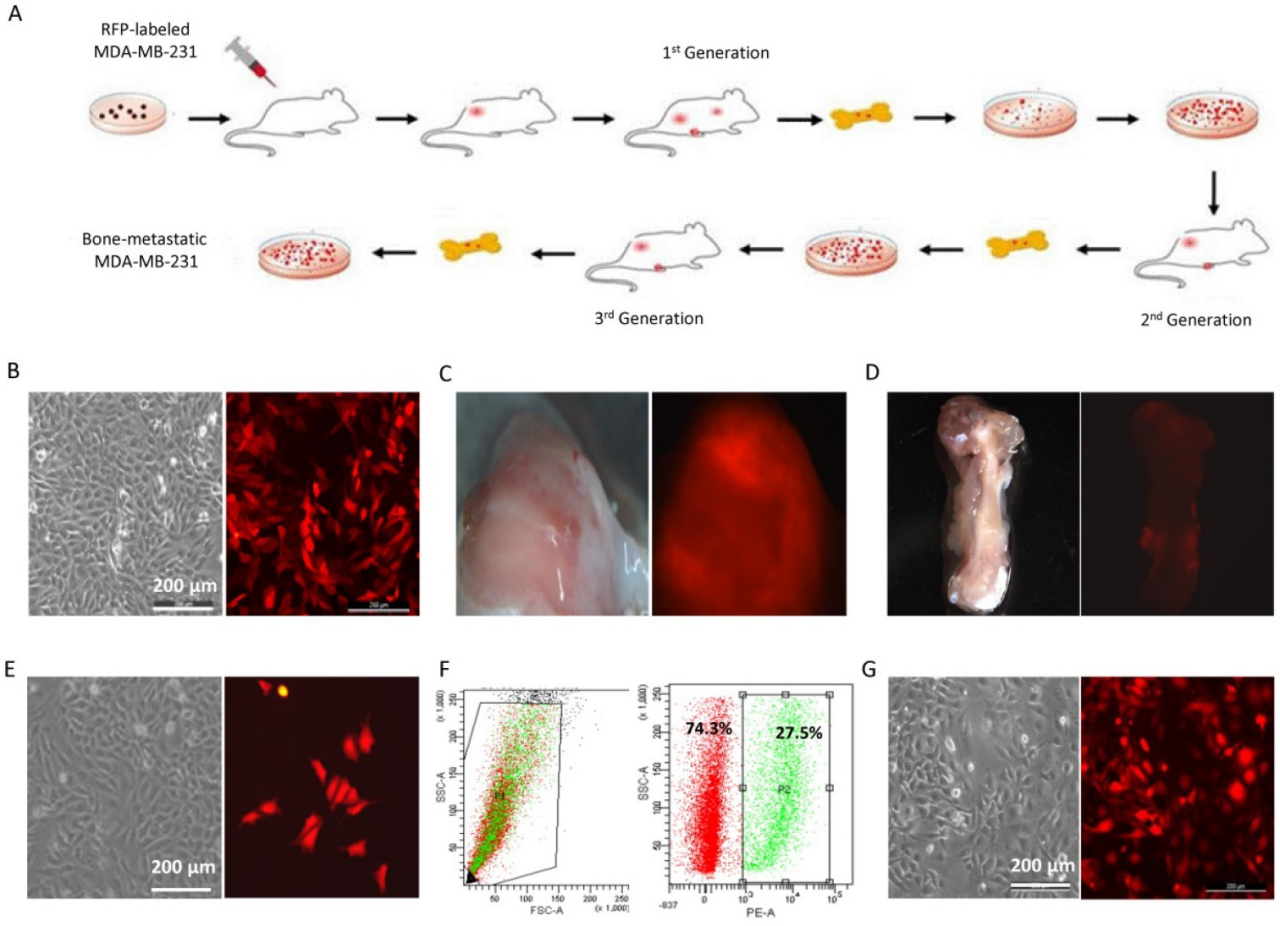
Establishment of bone-metastatic cell sublines BM1 and BM2 from MDA-MB-231. **A:** Schematic representation of the procedure to establish bone-metastatic cell sublines from MDA-MB-231. **B:** Brightfield (left) and fluorescence (right) images of MDA-MB-231 cells traduced with TurboRFP lentivirus. **C:** Brightfield (left) and fluorescence (right) images of mouse breast tumors grown from RFP-MDA-MB-231 cells. **D:** Brightfield (left) and fluorescence (right) images of mouse shinbone from tumor-burden mouse. **E:** Brightfield (left) and fluorescence (right) images of cells from bone marrow of tumor-burden mouse. **F:** RFP positive cell sorted using flow cytometry. **G:** Brightfield (left) and fluorescence (right) images of purified bone-metastatic cells derived from MDA-MB-231.

In order to determine the mechanism in regulating bone metastasis of breast cancer, bone-metastatic MDA-MB-231 cell sublines were established by cell sorting from the bone of nude mouse carrying mammary gland tumors generated by transplantation of RFP-MDA-MB-231 cells. The cell transplantation into the mammary gland and metastatic cell isolation from the bone were performed three rounds as shown in Figure 1A. MDA-MB-231 cells were transduced with RFP (Figure 1B), and transplanted into the fourth fat pad of nude mouse to generate breast tumors (Figure 1C). In 6 weeks after cell transplantation, the mouse was sacrificed and the femora and shinbone from hind legs were collected (Figure 1D). Cell mixture from bone marrow was cultured* in vitro*, in which metastatic cancer cells with RFP fluorescence were observed (Figure 1E). After cell sorting with flow cytometry, the RFP positive cells representing metastatic breast cancer cells were purified from the bone (Figure 1F). Through three rounds of screening, two bone metastatic breast cancer cell sublines with RFP positive were derived from MDA-MB-231 cells in different individual mouse, and named BM1 and BM2, respectively (Figure 1G).

### *In vivo* validation of the metastatic ability of BM1 and BM2

**Figure 2:**
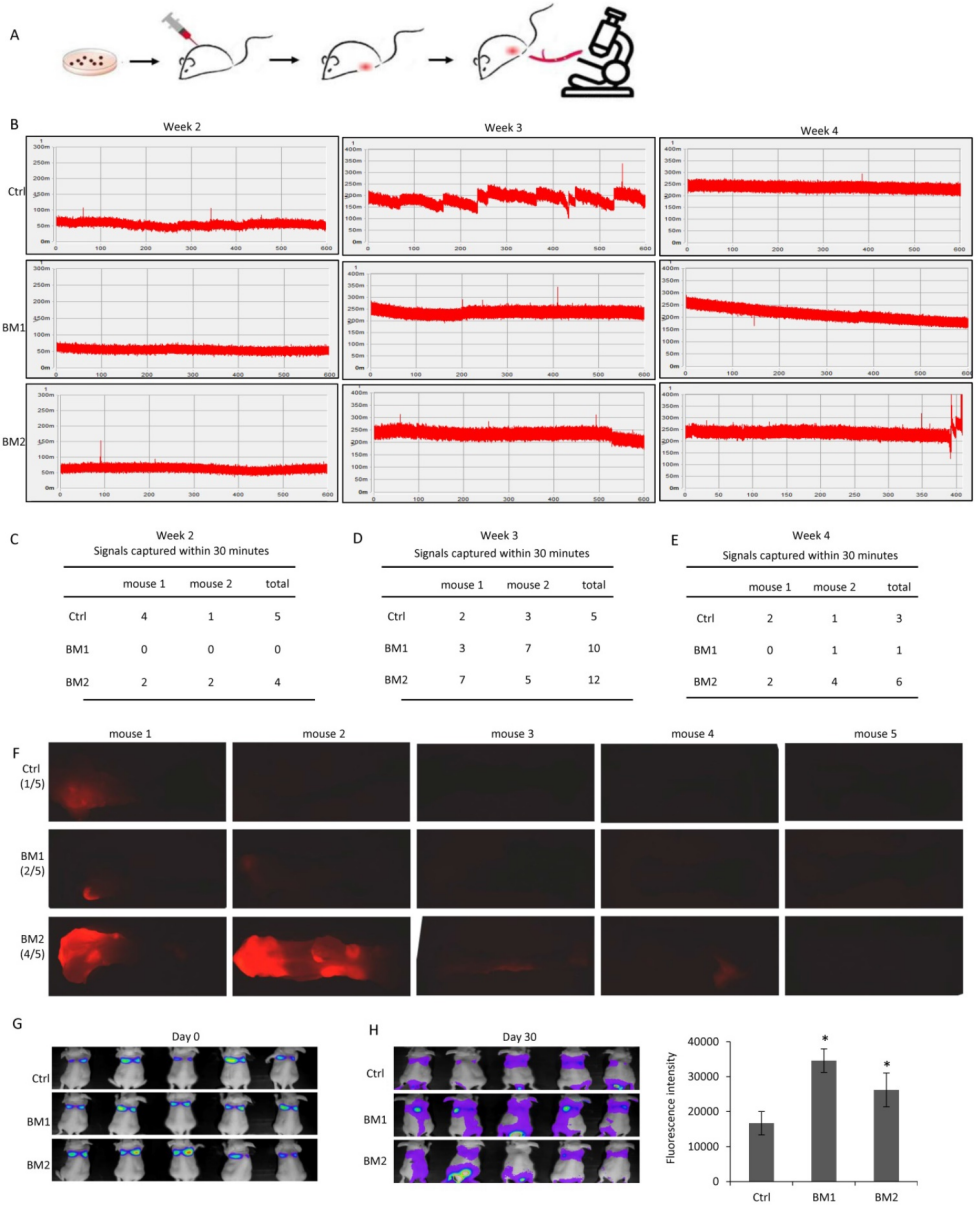
***In vivo* validation of the bone metastatic ability of BM1 and BM2. A:** Schematic representation of the procedure to track RFP positive BM1 and BM2 cells invaded into circulation using *in vivo* flow cytometry. **B:** Representative records of the RFP fluorescence signals captured by *in vivo* flow cytometry system when the circulating BM1, BM2 or control cells passed through the artery. The detection was performed at week 2, week 3 and week 4 after cell transplantation into the fat pad of mice (n=2 in each group). RFP-MDA-MB-231 cells served as control. **C-E:** Quantitative analysis of fluorescence signals captured at week 2 (C), week 3 (D), and week 4 (E). **F:** Breast tumor-burden nude mice were prepared by transplantation of 1×10^6^ BM1, BM2 or MDA-MB-231 control cells into the fat pad of the mammary gland. After sacrifice in 4 weeks, bones from hind legs were applied to a fluorescence dissect microscope detecting cancer cells metastasized to the bone. 1 from 5 mice in RFP-MDA-MB-231 control group, 2 from 5 mice in BM1 group, and 4 from 5 mice in BM2 group showed RFP fluorescence signals. **G,H:** *In vivo* images showing the florescence signals in the mice at day 0 (G) and day 30 (H) after tail-vein injection with 1x10^6^ Firefly luciferase-transduced BM1, BM2 or control cells to each mouse (n=5 in each group).

Before functional analysis with BM1 and BM2 cell sublines, their metastasis ability was examined* in vivo*. *In vivo* flow cytometry was applied to the mice carrying breast tumors grown from the transplantation of BM1 or BM2, for the real-time detection of RFP positive metastatic tumor cells in circulation. As shown in Figure 2A, an artery at the ear of the mouse was chosen for data acquisition. The laser slit can capture the RFP fluorescence signal in the circulating BM1/BM2 cells when passing through the artery. The detection was performed at week 2, week 3 and week 4 after cell transplantation. The fluorescence signals in circulating cells were shown in Figure 2B. Interestingly, quantitative analysis indicated general amount of circulating BM1/BM2 signals at week 2 (Figure 2C), much more signals at week 3 (Figure 2D), and dropped back to general level at week 4 (Figure 2E), compared to control MDA-MB-231 cells. Although this kind of pattern needs to be double-confirmed using other approaches, such as fluorescence diffuse optical tomography, the trend of Normal Distribution-like pattern of circulating tumor cell numbers is beyond our understanding that cancer metastatic grade may correlate with tumor size and cancer progression.

In order to further validate bone metastasis of BM1 and BM2 *in vivo*, breast tumor-burden nude mice were prepared by cell transplantation to the fat pad of mammary gland. All mice were sacrificed in 4 weeks, bones were collected from hind legs after removing skeletal muscle and vessels, and applied to a fluorescence dissect microscope for detection of RFP signals from the cancer cells metastasized to the bone. As seen the bone images in Supplemental Figure S1 (brightfield) and Figure 2F (fluorescence), 2 from 5 mice in BM1 group, and 4 from 5 mice in BM2 group showed clear red fluorescence signals, compared to 1 from 5 mice in RFP-MDA-MB-231 control group (Figure 2F).

In addition, a firefly luciferase vector was transduced into BM1 and BM2 cells, respectively, followed by a tail-vein injection into nude mice. As *in vivo* images shown in Figure 2G, at the day of cell injection most of the cells enriched in the lung. The florescence intensities did not show the difference between control and BM1/BM2 cells. However, in 30 days after cell injection, the *in vivo* images showed significantly stronger florescence signals in the bone of spine and hind legs of both BM1 and BM2 mice compared to control (Figure 2H).

### Oncogenic validation of BM1 and BM2 in cellular proliferation, migration, invasion and EMT

**Figure 3:**
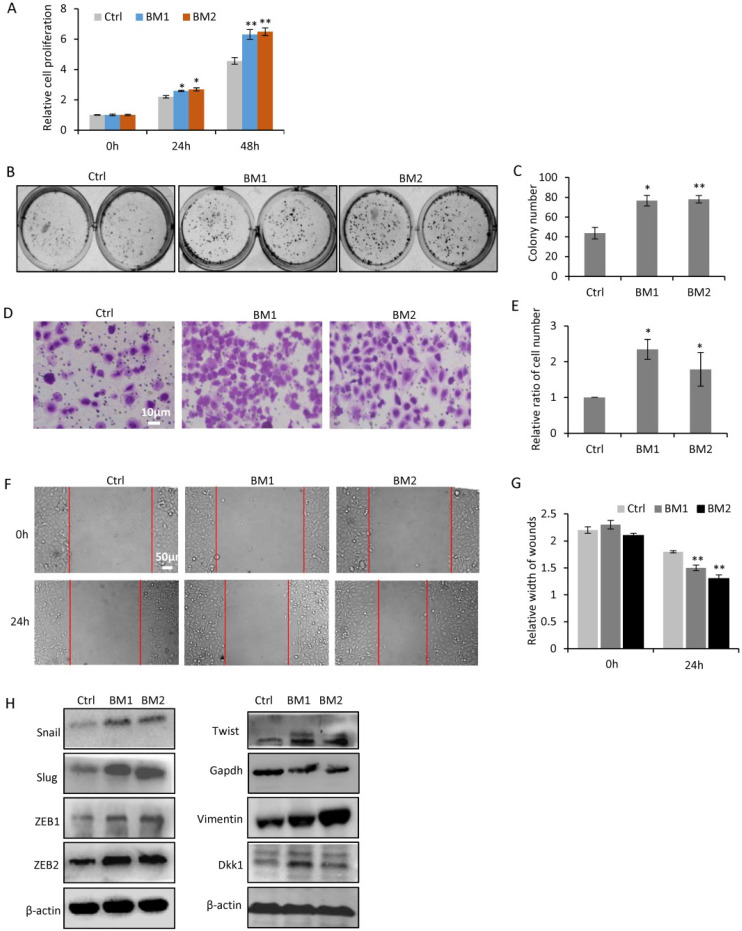
** Oncogenic validation of BM1 and BM2. A,B:** Cell proliferation assays by CCK8 (A) and Colony formation (B) indicated the increased ability to proliferate of BM1 and BM2, compared to MDA-MB-231 cells. **C:** Quantitative analysis of B. **D:** Transwell assay demonstrated the increased ability to invade of BM1 and BM2, compared to MDA-MB-231 cells. **E:** Quantitative analysis of D. **F:** Wound healing assay demonstrated the increased ability to migrate of BM1 and BM2, compared to MDA-MB-231 cells. **G:** Quantitative analysis of F. **H:** Western blot demonstrated the higher expression levels of EMT markers including Snail1, Slug, ZEB1, ZEB2, Twist, Vimentin, and DKK1 in both BM1 and BM2, compared to MDA-MB-231 cells. β-actin and GAPDH served as loading control. Values are equal to mean ± SEM. *p<0.05, **p<0.01.

In order to determine the oncogenic properties of BM1 and BM2 sublines, cell proliferation assay (Figure 3A) and colony formation assay (Figure 3B, 3C) demonstrated higher rate of cell proliferation in BM1 and BM2 than that in MDA-MB-231 cells. Transwell (Figure 3D, 3E) and wound healing (Figure 3F, 3G) assays demonstrated increased cell migration and invasion of BM1 and BM2, compared to MDA-MB-231 cells. The expression levels of EMT markers including Snail1, Slug, ZEB1, ZEB2, Twist, and Vimentin showed significantly increase in both BM1 and BM2 (Figure 3H). In addition, DKK1, an inducer of bone metastasis and inhibitor of lung metastasis from breast cancer 16, also showed upregulation in BM1 and BM2 (Figure 3H).

### Cancer stem cell-like properties in BM1 and BM2 cells

**Figure 4:**
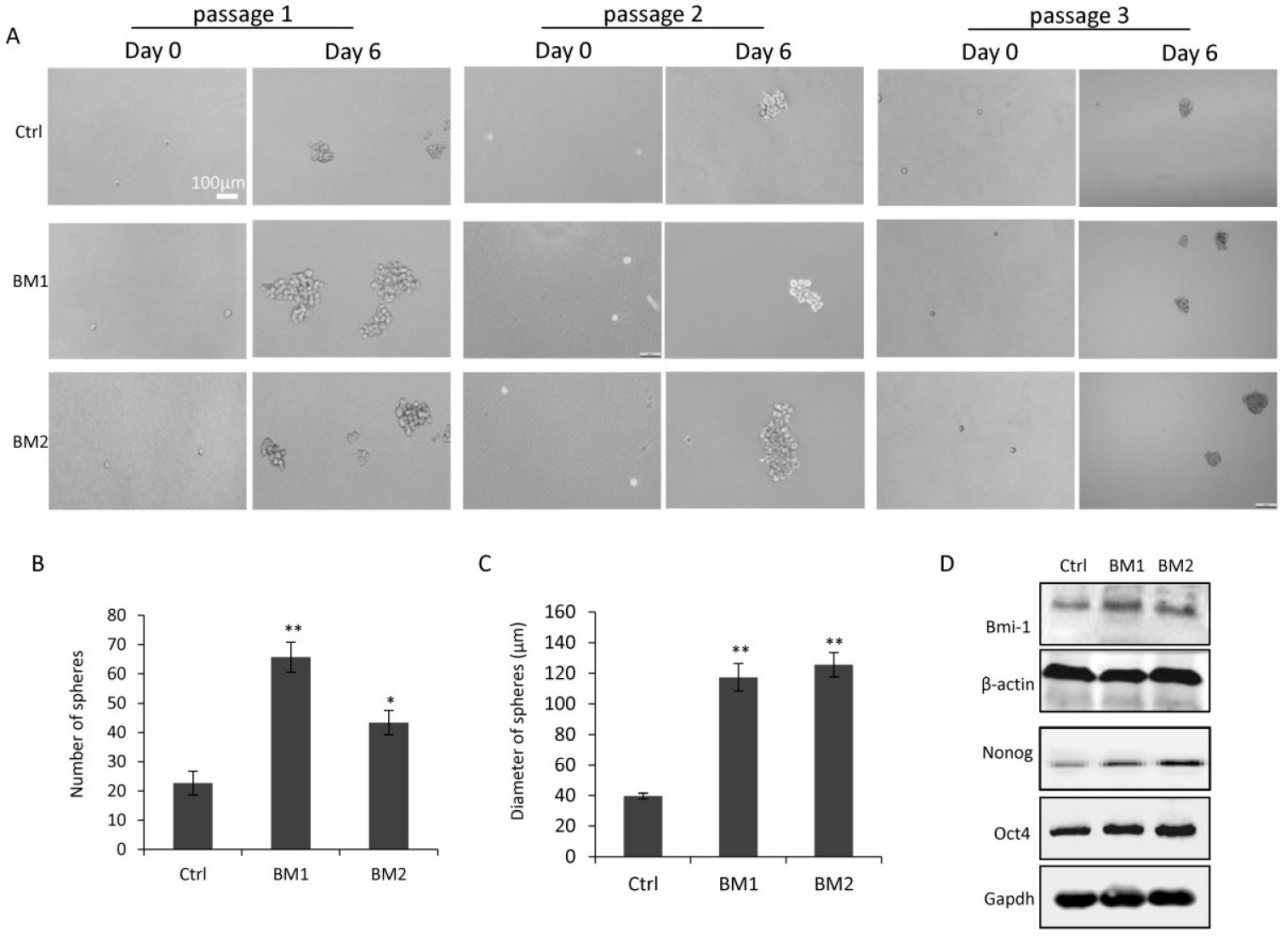
** Cancer stem cell-like properties in BM1 and BM2. A:** Mammosphere assays were applied to BM1, BM2 and MDA-MB-231 cells for three passages in serum-free culturing medium to validate the stemness of the cell sublines. **B,C:** Quantitative analysis for the number (B) and size (C) of the spheres formed in the third passage of A. **D:** Western blot analysis for the expression level of stemness markers including Bmi-1, Oct4, and Nanog in BM1, BM2 and MDA-MB-231 cells. β-actin and GAPDH served as loading controls. Values are equal to mean ± SEM. *p<0.05, **p<0.01.

In view of the correlation between stemness and metastasis in breast cancer cells, mammosphere assays were applied to BM1 and BM2 cells to determine how close they are to cancer stem cells. As shown in Figure 4A, after 6 days of culturing under serum-free condition, spheres were formed from survived cells which represent cancer stem cells. Notably, the assays were performed for 3 passages, demonstrating the inheritable self-renewal and mammosphere-formation abilities of BM1 and BM2 cells. Quantitative analysis indicated a significant increase in both number and size of the spheres formed from BM1 and BM2 sublines, compare to MDA-MB-231 control cells (Figure 4B and 4C). Western blot analysis on the stemness markers including BMI-1, OCT4, and Nanog indicated upregulation in both BM1 and BM2 cells, compared to MDA-MB-231 control cells (Figure 4D).

### Tumorigenesis of BM1 and BM2 cells *in vivo*

**Figure 5:**
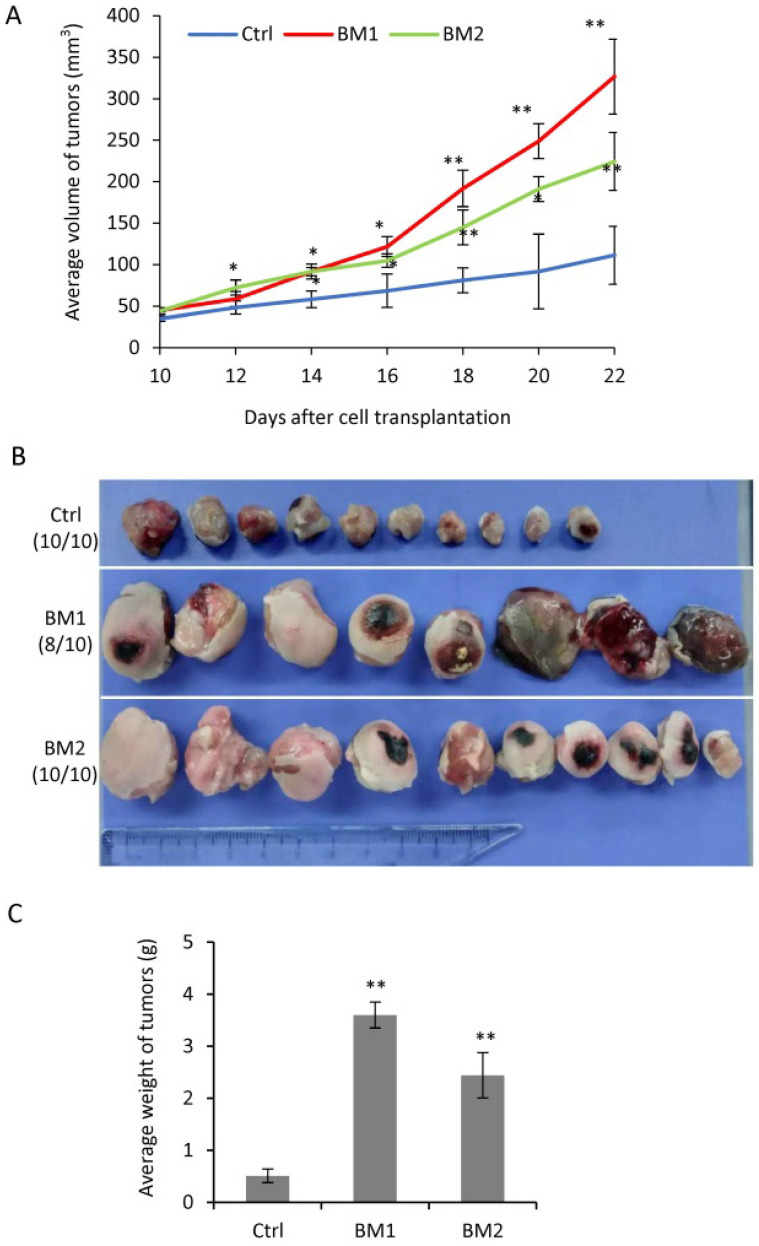
** Tumorigenesis of BM1 and BM2 *in vivo.* A:** Tumor growth curve by transplantation of BM1, BM2 or MDA-MB-231 control cells into the fat pad of nude mice (n=10 in each group). **B:** Tumor photos in three groups in A. **C:** Average weight of tumors in three groups in A. Values are equal to mean ± SEM. *p<0.05, **p<0.01.

In order to determine the tumorigenic ability of BM1 and BM2 cells *in vivo*, immunodeficient nude mice was used to grow tumors by cell transplantation into the fat pad of the mammary gland. From day 10 on after cell transplantation, the tumors were measured every other day until day 22 when all mice were sacrificed. The tumors grown from BM1 and BM2 cells both showed faster in growth (Figure 5A), larger in size (Figure 5B) and heavier in weight (Figure 5C), compared to MDA-MB-231 control.

### Gene signature in BM1 and BM2 cells

**Figure 6:**
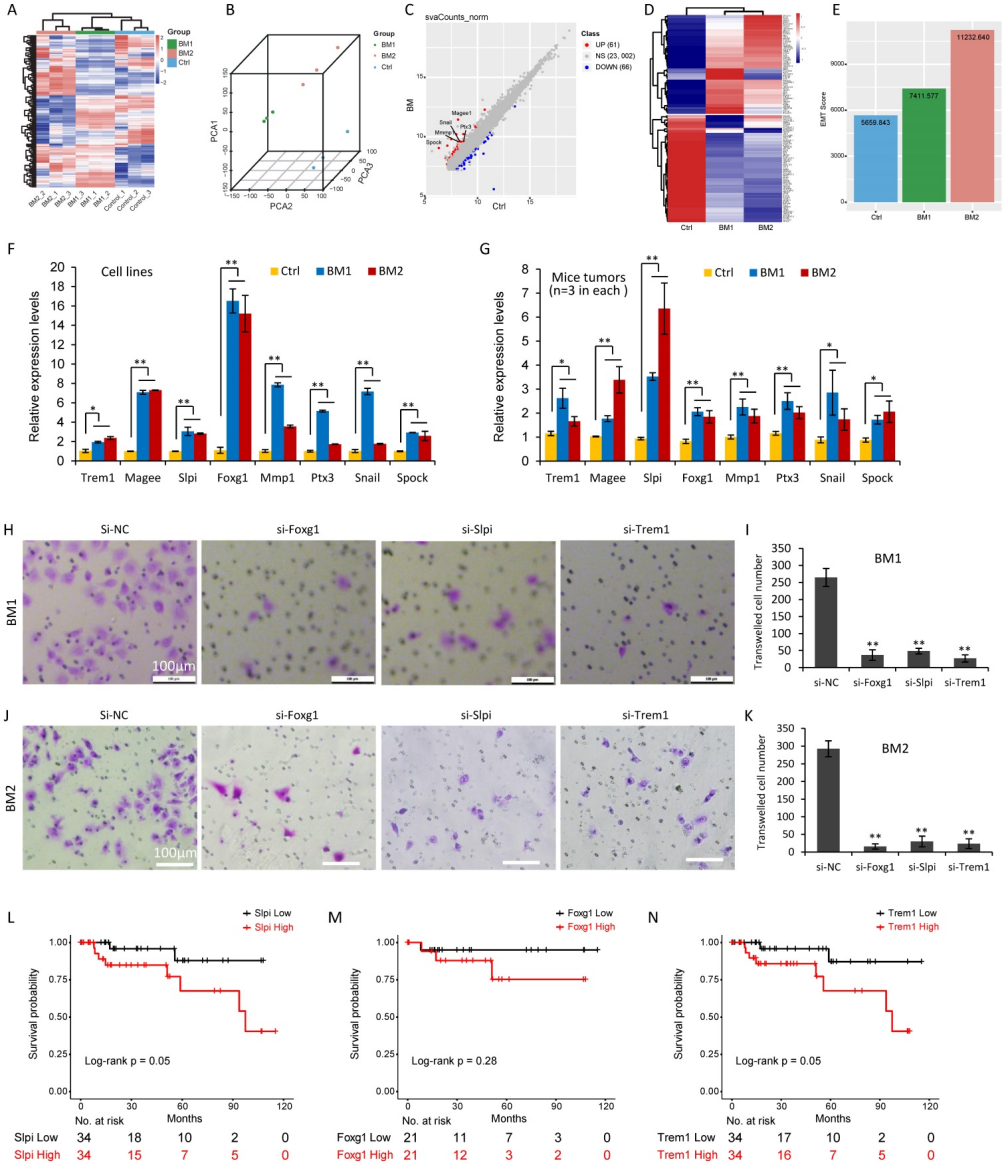
** Deep sequencing analysis identified a gene signature in BM1 and BM2 cells. A:** Heatmap of gene expression profiling in BM1, BM2 or MDA-MB-231 control cells (triplicates in each group) using hierarchical clustering algorithm. **B:** Principal component analysis (PCA) clustered the nine samples in three groups with three-dimensional (3D) space. **C:** DESeq2 analysis identified 127 differently expressed genes (DEGs) in both BM1 and BM2 cells compared to MDA-MB-231 control, including 61 upregulated genes and 66 downregulated genes. Representative EMT markers were marked with gene symbols. Differentially expressed gened (DEGs) were defined by fold change (FC) ≥ 2 and a false discovery rate (FDR) < 0.05. **D:** Heatmap for expression pattern of the 127 DEGs in BM1, BM2 and MDA-MB-231 control cells. **E:** Bar graphs showing EMT score derived from the expression levels of EMT signature genes in BM1, BM2 and MDA-MB-231 control cells. **F, G:** Quantitative RT-PCR analysis was applied to confirm the expression pattern of 8 representative genes in BM1, BM2 and MDA-MB-231 control cells (F), or mice tumors (n=3 in each group) derived from the BM1, BM2 and control cell transplantation (G). **H-K:** Cell invasion analysis on BM1 (H,I) and BM2 (J,K) cells after siRNA knockdown of Foxg1, Slpi, and Trem1, respectively. Scale bar=100 µm**. L-N:** Overall survival analysis on 68 TNBC patients with high or low expression levels of Slpi (L), Foxg1 (M), and Trem1 (N) using Log-Rank tests. Values are equal to mean ± SEM. *p<0.05, **p<0.01.

In order to determine the gene expression signature in the bone-metastatic breast cancer cells, total RNAs from MDA-MB-231 cells (Control) and BM1 and BM2 cells were applied for deep-sequencing analysis (Supplemental Table S1). Agilent 2100 Bioanalyzer was used for quality validation. Totally 19,113 genes were analyzed with diverse expression levels over the three groups, and standard deviation (SD) greater than 1. A hierarchical clustering algorithm was used to group samples on the basis of expression similarity over these genes (Figure 6A). As expected, three replicates in each group shared the similar gene expression profile and were clustered together (Figure 6A). The distribution of samples in three-dimensional (3D) space determined by principal component analysis (PCA) further demonstrated the consistency in gene expression of three biological replicates in each group, which were clustered together (Figure 6B).

To find out the differently expressed genes (DEGs) in BM1 and BM2, DESeq2 analysis identified 127 DEGs in both BM1 and BM2 cells compared to control, in which 61 genes showed upregulation and 66 genes showed downregulation (Supplemental Table S2-S3, Figure 6C), with fold change (FC) ≥ 2 and a false discovery rate (FDR) < 0.05. Notably, a subset of EMT-related genes including Mmp1, Magee1, Snail2, Spock1 and Ptx3, were significantly upregulated in BM1/BM2 cells with FC 22.0, 3.3, 2.8, 2.6 and 2.1, respectively (Figure 6C). Expression pattern of the 127 DEGs in BM1, BM2 and MDA-MB-231 control cells were shown in Figure 6D and Supplemental Figure S2. In addition, EMT score, which was derived from the expression levels of EMT signature genes, further demonstrated higher level in both BM1 and BM2 sublines compared to MDA-MB-231 control cells (Figure 6E).

In order to validate the gene expression signature obtained from deep-sequencing analysis, quantitative RT-PCR analysis was applied to 8 representative EMT genes including Mmp1, Magee1, Snail, Spock1, Ptx3, Trem1, Slpi, and Foxg1. Both cell lines (Figure 6F) and mice tumors (Figure 6G) derived from BM1 and BM2 transplantation showed higher expression levels of the 8 genes compared to MDA-MB-231 control, which is consistent with the deep-sequencing data.

In order to further reveal the function of the genes identified in the current study, Foxg1, Trem1 and Slpi were selected for functional analysis. This is the first report for the upregulation of these genes in the bone-metastatic breast cancer cells. SiRNAs targeting these genes were transfected to BM1 and BM2 cells, followed by transwell cell invasion analysis (Figure 6H-6K). Three siRNA sequences were tested for each gene. Foxg1-siRNA1, Trem1-siRNA1 and Slpi-siRNA1 showed 60-80% gene knockdown efficiency (Supplemental Figure S3). As such, they were selected for functional analysis. Cell invasive abilities of both BM1 and BM2 dramatically decreased after knockdown of Foxg1, Slpi or Trem1, respectively. (Figure 6H-6K). The survival analysis indicated the poor survival associated with high expression levels of Slpi and Trem1 in TNBC with statistically significance (Figure 6L-N). Foxg1 showed a similar trend, but further analysis using a larger database of patients is required to determine the correlation between the expression level of Foxg1 and 10-year survival rate.

### Upregulated signaling pathways in BM1 and BM2 cells

**Figure 7:**
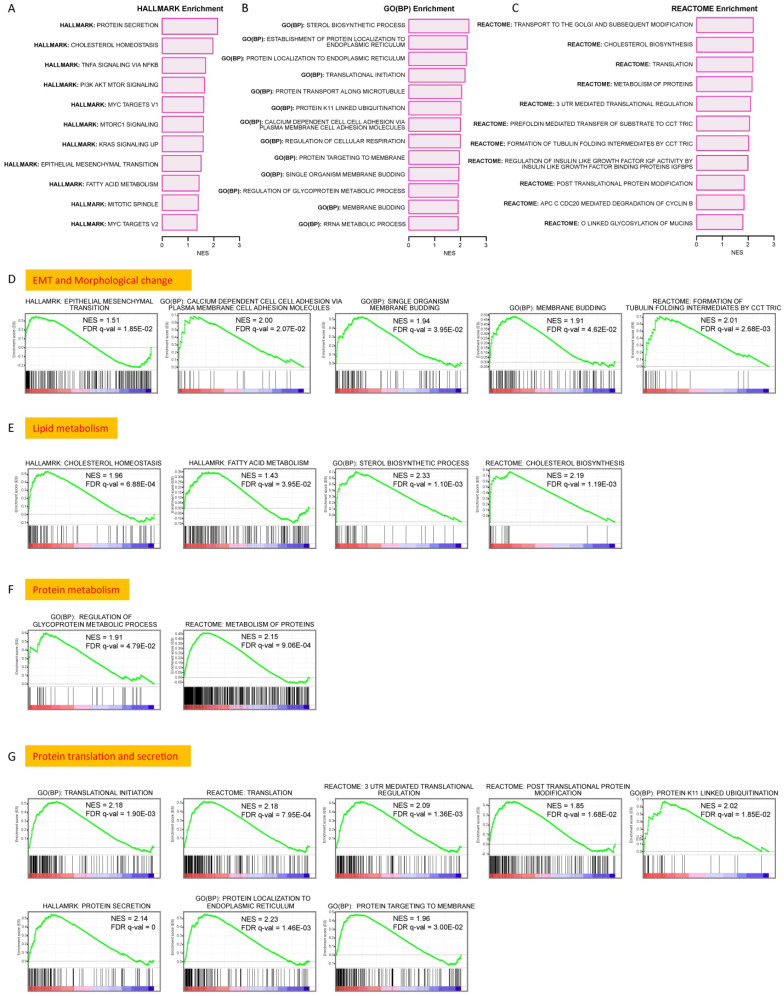
** Upregulated signaling pathways in BM1 and BM2 cells. A-C:** Upregulated signaling pathways by gene set enrichment analysis (GSEA) of the 127 DEGs using databases of Cancer Hallmark (A), GO (BP) (B) and Reactome (C). **D:** GSEA analysis showing significant upregulation of pathways in regulation of EMT, cell membrane budding and morphologic change. **E:** GSEA analysis showing significant upregulation of pathways in regulation of lipid metabolism including cholesterol biosynthesis/homeostasis and fatty acid metabolism. **F:** GSEA analysis showing significant upregulation of pathways in regulation of protein metabolism. **G:** GSEA analysis showing significant upregulation of pathways in regulation of protein biosynthesis and secretion. The significant enrichment of a gene set was defined by FDR < 0.05.

Gene set enrichment analysis (GSEA) was performed using databases of Cancer Hallmark, GO (BP) and Reactome to determine regulatory pathways the DEGs may involve. A series of gene sets showed significant enrichment (FDR < 0.05) in multiple pathways. The upregulated gene sets in Cancer Hallmark (Figure 7A, Supplemental Figure S4A), GO(BP) (Figure 7B, Supplemental Figure S4B) and Reactome (Figure 7C, Supplemental Figure S4C) strongly suggest the involvement of the DEGs in pathways regulating EMT, cell membrane budding and morphological changes (Figure 7D), lipid metabolism (Figure 7E), protein metabolism (Figure 7F), and gene translation & protein secretion (Figure 7G). Notably, multiple oncogenesis-related signaling pathways, such as PI3K/AKT/mTOR signaling, Myc signaling, Kras signaling, et al., showed significant upregulation (Supplemental Figure S5). In addition, a few enriched gene sets showed downregulation in GSEA analysis, which were shown in Supplemental Figure S6A-S6C.

## Discussion

It has been well recognized that the majority of the death from breast cancer is due to metastasis of cancer cells to distant organs. Although emerging findings indicate the microenvironment in the host organs playing important roles in survival, seeding and tumor regeneration, the metastatic cancer cells are still the driving force of metastasis 17,18. Literature has identified a subset of host organ-related genes and/or molecules in regulating breast cancer metastasis 10,12,16,19. However, the mechanism in regulating the selection of distant organs for breast cancer metastasis remains unclear partly due to lacking of a gene signature to distinguish cancer cells with different preference of metastatic organs. Consequently, there is still no strategy developed to prevent and treat metastasis of breast cancer.

Although previous study by Kang et al. 10 had derived multiple osteolytic bone metastatic sublines including 1833 and 1834 from MDA-MB-231 cells, and identified an important gene signature encoding osteolytic and angiogenic factors to promote tumor cell metastasis to the bone, there are still several limitation and shortages. For example, the approach making bone-metastatic mice model by cardiac ventricle injection of MDA-MB-231 cells may not perfectly mimic the bone metastasis from primary tumor at the site of breast in patients. In the work, tumor cells isolated from osteolytic bone lesions using X-ray imaging and different attachment to culturing plate may be mixed with other cell types, such as immune cells. As compensation to the work, we herein transplanted florescence-labeled MDA-MB-231 cells to the fat pad of nude mice to generate breast tumors, tracked the tumor cells invaded into the circulation by *in vivo* flow cytometry system, and isolated metastatic tumor cells from the bone by FACS cell sorting. Eventually, we established two cell sublines with bone-metastatic preference. As well-determined by literature 10,20, bone-metastatic clones from MDA-MB-231 cells perform osteolytic bone metastasis. The same to BM1 and BM2 sublines. We believe these sublines will be regarded as an important cell model not only for studying cell fate determination of metastatic breast cancer cells, but also for developing therapeutics to inhibit bone metastasis in breast cancer.

Functional studies* in vitro* and *in vivo* both demonstrated the high degree of malignancy of the two bone-metastatic cell sublines, including increased abilities in cell proliferation, migration, invasion, EMT and tumor regeneration. Notably, the bone-metastatic cells showed more cancer stem cell-like characteristics, such as higher percentage of stem cell subpopulation, more chance to form mammospheres under serum-free culturing condition, and higher expression levels of stemness genes. We believe it is the high degrees of malignancy and stemness in these cells that are mainly responsible for the resistance to therapy, metastasis and recurrence. Administration of therapeutics targeting these cells may hold promise in treatment of metastatic breast cancer.

It is of great interest that we are the first to find a Normal Distribution-like pattern of the bone-metastatic breast cancer cells invading into circulation as shown in Figure 2B. *In vivo* tracking system on living mouse indicated a regular amount of circulating tumor cells in the early and late stages during breast tumorigenesis, while a peak level of ~3 times more than control of circulating tumor cell signals were recorded at the middle stage of tumor growth. It suggested that the cell metastasis is not dependent on the tumor size and tumor progression, which differs from our conventional understanding for metastasis. Although this novel observation needs further evaluation, it provides encouragement to pay more attention to the stage during tumor growth when developing a strategy in treatment of metastatic breast cancer.

As expected, RNA deep sequencing analysis on the bone-metastatic cell sublines identified a subset of gene signature, and confirmed the upregulation of genes involved in the signaling pathways in regulating EMT, membrane budding, and cellular cytoskeleton and morphology. A group of EMT-regulating genes including Fgf5 and Mmp1 showed significant upregulation in BM1 and BM2 cells, which is consistent with the gene signature in the bone-metastatic 1833 cell subline 10. Garcia T. et al. isolated an isogenic B02 clone from MDA-MB-231 cells with unique ability to form rapidly growing osteolytic bone metastases 20. Using whole transcriptomic analysis they identified a gene signature in B02 cells. In consistence, subsets of genes were overlapped between the gene signatures in B02 cells and BM1/BM2 cells, such as Nap1l3 and Mmp1 (2 of the top 5 upregulated genes in BM1/BM2 (Supplemental Table S2). However, a few genes showed opposite expression trend, such as Cyp1b1 and HLA-DPA1, which increased in BM1/BM2 cells while decreased in B02 cells 20. Although Cyp1b1 has been reported to promote breast cancer metastasis 22, more functional validation both *in vitro* and *in vivo* is still required. Additional genes, such as Foxg1, Trem1, Slpi, et al., were only identified by our current study with upregulation in the bone-metastatic breast cancer cells. A recent publication reported Foxg1 promotes hepatocellular carcinoma EMT by activating Wnt signaling 21. Trem1 has been expected to be a prognostic marker for detection of lung metastases from solid tumors 23. Slpi was considered as a potential target for inhibiting metastasis of triple-negative breast cancers 24. A highly metastatic triple-negative breast cancer 4T1 cells secret more Slpi than non-metastatic counterparts 24. The functional analysis of the three genes *in vitro* demonstrated a dramatic increase in cell invasive ability of BM1 and BM2 cells (Figure 6H-6K).

We also found a subset of genes involved in signaling pathways upregulated in BM1 and BM2 cell sublines, such as mTOR signaling, Myc signaling, protein translation, peptide elongation, mRNA 3'UTR regulation, protein secretion, and cell surface protein targeting to cell membrane, which are consistent with literature that metastatic cancer cells not only showed activation of oncogenic signaling including c-myc/β-catenin signaling and PI3K/AKT/mTOR signaling 25-27, but also showed high levels of secretary or cell surface proteins in expression 10, involving in cell homing to bone, angiogenesis, invasion, and osteoclast recruitment 10. Furthermore, based on analysis of multiple publicly available datasets, a positive association between the gene sets representing protein translation and metastasis in basal-like breast cancer has been reported 28. As such, the posttranscriptional regulation and protein translation/secretion must be of help in synthesizing adequate metabolic enzymes and structural proteins in the cells undergoing EMT, invasion and metastasis.

Interestingly, the current study found that lipid metabolism-related pathways, including cholesterol biosynthesis, sterol biosynthesis, and fatty acids metabolism, showed upregulation in the bone metastatic breast cancer cell sublines, demonstrating the additional energy requirement for cancer cell metastasis to distant organs. Moreover, the current finding may suggest that the energy required for breast cancer cells metastasis to the bone is mainly from lipolysis of sterol and fatty acid, although more evidences are required for further validation.

## Conclusions

Two bone-metastatic cell sublines were derived from triple-negative breast cancer cell MDA-MB-231, which exhibit higher degree of stemness and malignancy than parental cells. Using the cell sublines we identified a gene signature in upregulating EMT, cell membrane budding, lipid metabolism and protein synthesis, which holds a potential to distinguish bone-metastatic breast cancer cells. These genes and pathways have potential as therapeutic targets in prevention of breast cancer metastasis to the bone.

## Supplementary Material

Supplementary figures.

Supplementary table S1.

Supplementary table S2.

Supplementary table S3.
