# Supplementary material for: Cancer stem cell property and gene signature in bone-metastatic Breast Cancer cells
Source: Int J Biol Sci. 2020 Jul 19;16(14):2580–94. doi: 10.7150/ijbs.45693 (PMC7415422; doi:10.7150/ijbs.45693)

Supplemental Information

Supplemental table S1: The number of reads of deep sequencing analysis of each sample.

Supplemental Table S2:Up-regulated genes in both BM1 and BM2

Supplemental Table S3: Down-regulated genes in both BM1 and BM2

Supplemental Figure S1: Images of bones taken under brightfield dissect microscope in breast tumor burden mice transplanted with BM1, BM2 or RFP-MDA-MB-231 control cells, corresponding to fluorescence images in Figure 2F.

Supplemental Figure S2: Figure 6D with a higher resolution. Heatmap for expression pattern of the 127 DEGs in BM1, BM2 and MDA-MB-231 control cells**.**

Supplemental Figure S3: Gene expression analysis to validate the knockdown effect of siRNAs targeting Foxg1, Trem1, and Slpi, respectively. Three siRNA sequences were applied for each gene. Values are equal to mean $\pm$ SEM. *p<0.05, **p<0.01.

Supplemental Figure S4: Additional pathways by GSEA analysis showing significant upregulation in BM1 and BM2, compared to MDA-MB-231 control cells. Databases of Cancer Hallmark (A), GO(BP) (B) and Reactome (C) were used for analysis.

Supplemental Figure S5: GSEA analysis demonstrated upregulation of oncogenic signaling pathways.

Supplemental Figure S6: Downregulated pathways identified by GSEA analysis using databases of Cancer Hallmark (A), GO(BP) (B) and Reactome (C).

Supplemental Figure S1

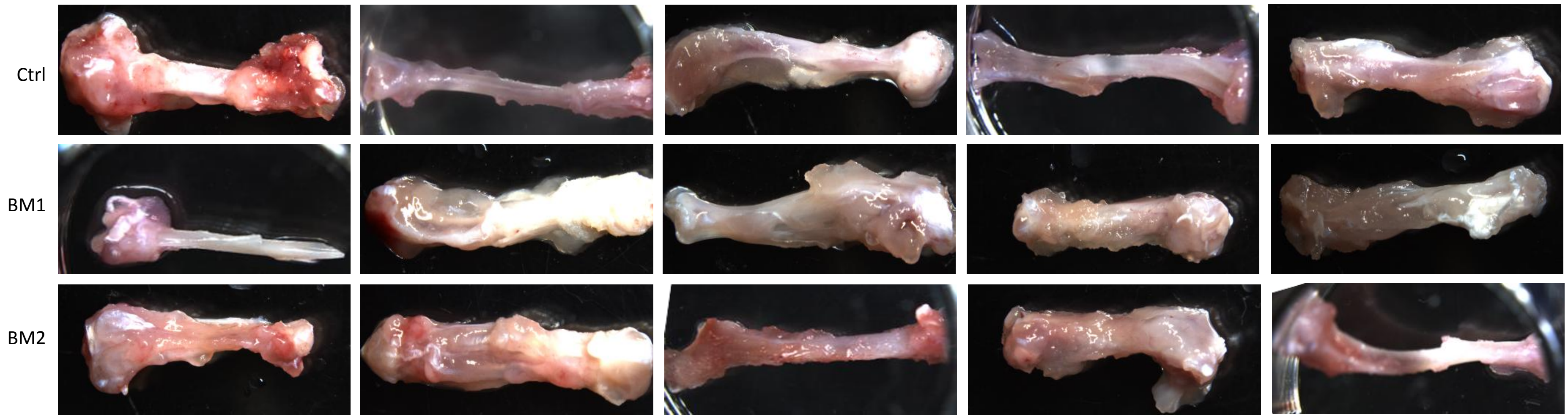

Supplemental Figure S2

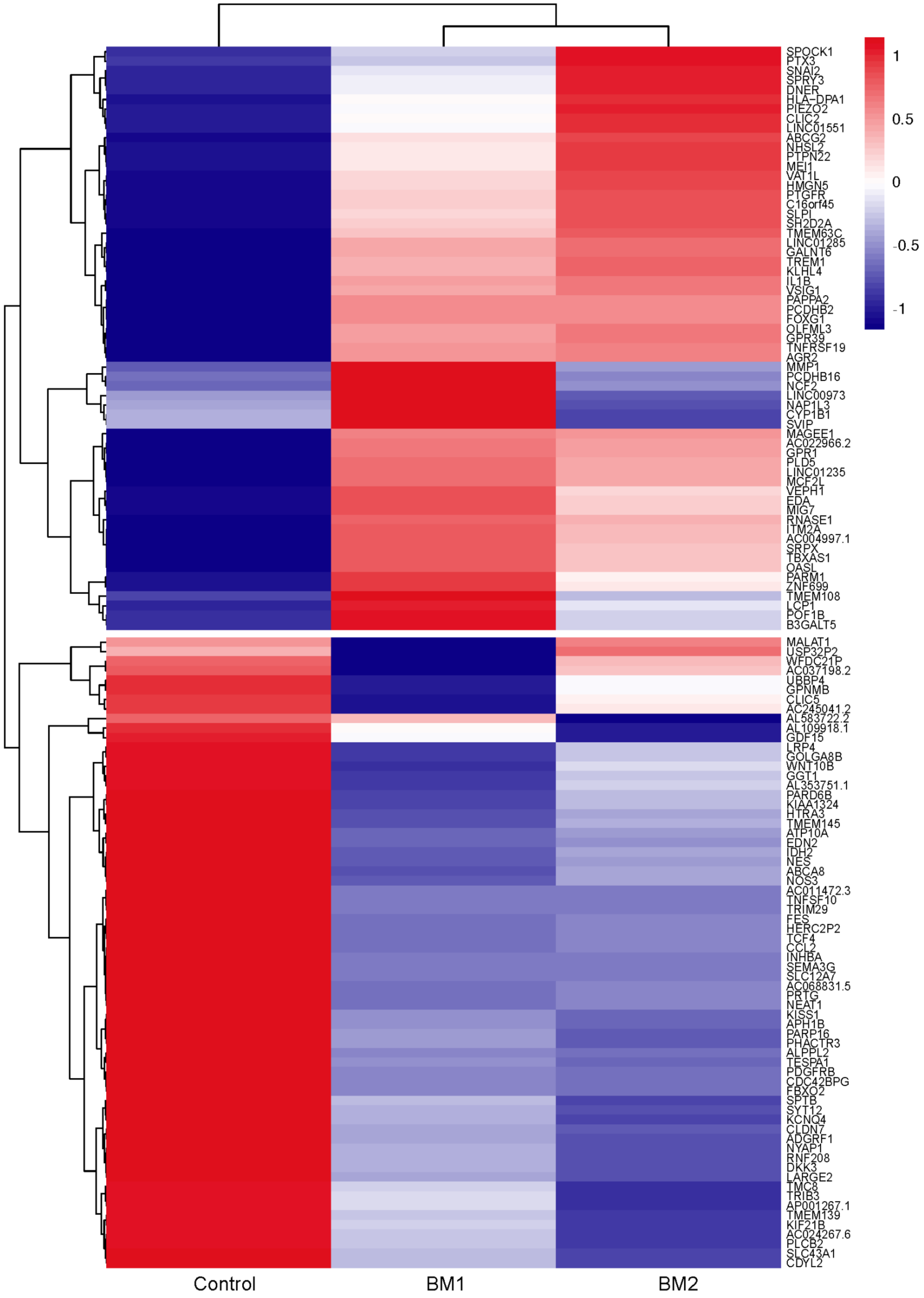

Supplemental Figure S3

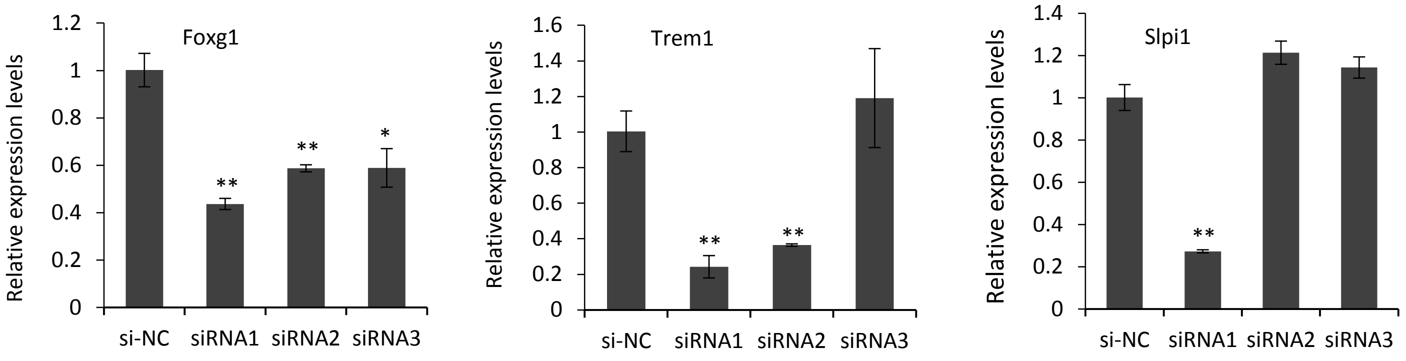

Supplemental Figure S4

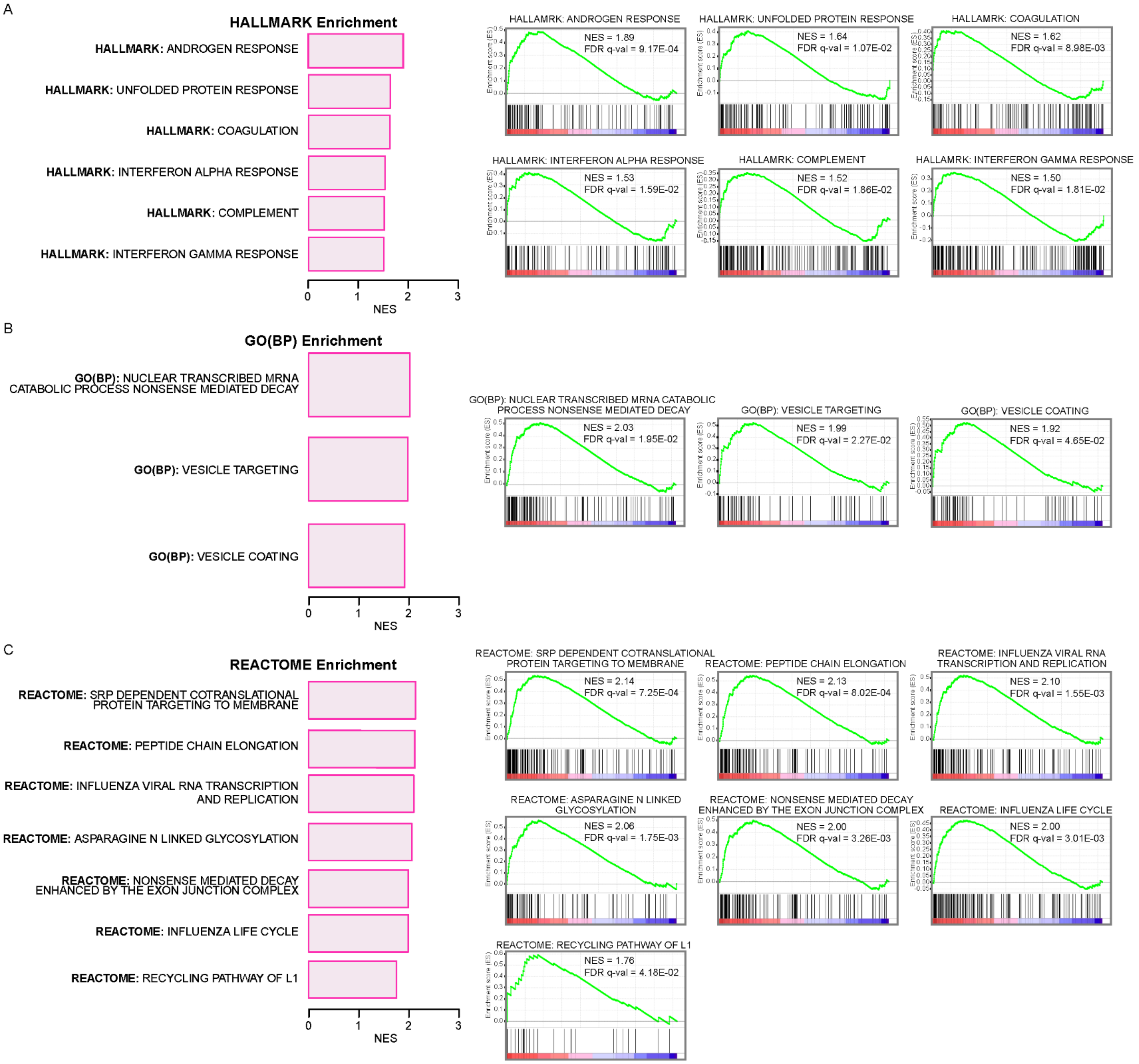

Supplemental Figure S5

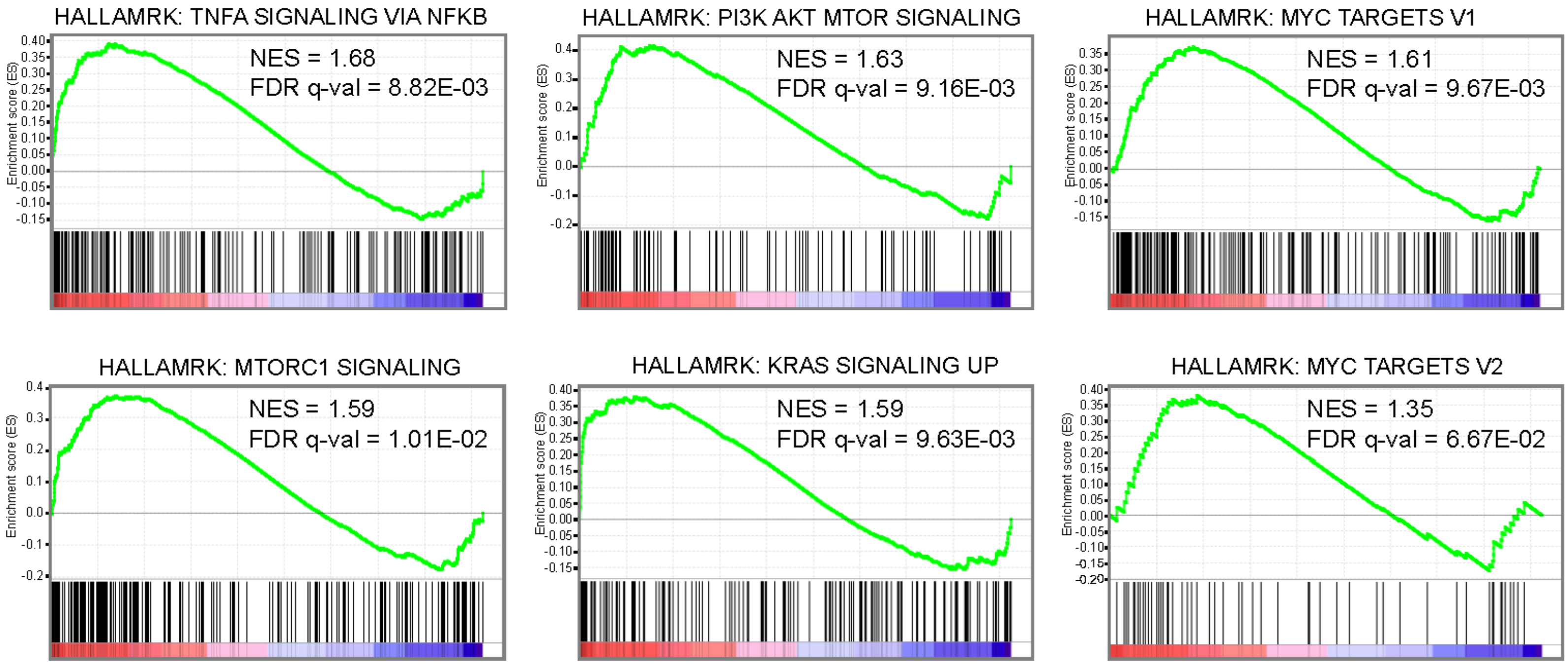

Supplemental Figure S6

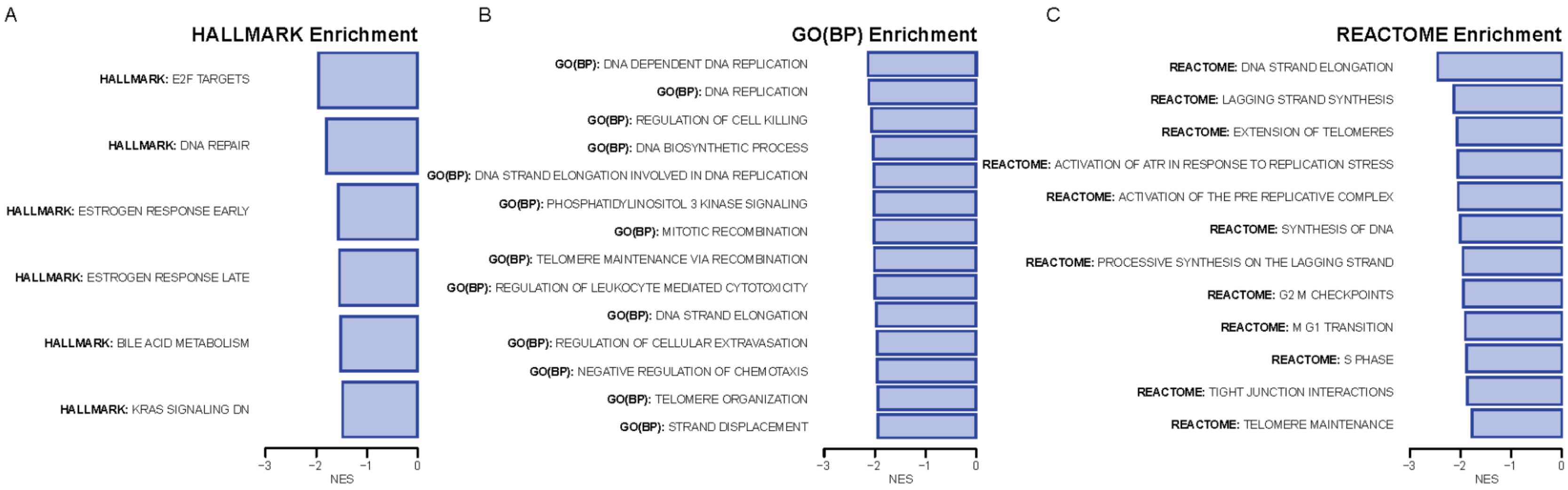

Supplement: Supplementary file 1 — Supplementary figures. [file ijbsv16p2580s1.pdf]
